# AP2M1 Amplification Orchestrates Notch‐Mediated Chemoresistance in Hematopoietic Stem Cells of Acute Myeloid Leukemia Patients

**DOI:** 10.1002/advs.202514566

**Published:** 2025-10-13

**Authors:** Hansong Lee, Eun Kyoung Kim, Hee Young Ju, Chae Rin Lee, Soyul Ahn, Eun‐Sun Kim, Kyungjae Myung, Won Kyu Kim, Su‐Yeon Cho, Yujin Kwon, Dokyoung Kim, Yeuni Yu, Eun Jung Kwon, Hyomin Kim, Seong Ik Mun, Dong Min Lim, Kihun Kim, Hye Jin Heo, Seung Eun Baek, Sun Young Lee, Hyunsoo Cho, Eun Young Choi, Tae Sik Goh, Ninib Baryawno, Dongjun Lee, Ki Sun Jung, Keon Hee Yoo, Chang‐Kyu Oh, Yun Hak Kim

**Affiliations:** ^1^ Medical Research Institute Pusan National University Yangsan 50612 Republic of Korea; ^2^ Medical Research Center for Bioreaction to Reactive Oxygen Species and Biomedical Science Institute School of Medicine, Core Research Institute (CRI) Kyung Hee University Seoul 02447 Republic of Korea; ^3^ Department of Pediatrics Samsung Medical Center Sungkyunkwan University School of Medicine Seoul 06351 Republic of Korea; ^4^ Department of Convergence Medical Sciences School of Medicine Pusan National University Yangsan 50612 Republic of Korea; ^5^ Center for Genomic Integrity Institute for Basic Science (IBS) Ulsan 44919 Republic of Korea; ^6^ Center for Natural Product Efficacy Optimization Korea Institute of Science and Technology (KIST) Gangneung 25451 Republic of Korea; ^7^ Department of Convergence Medicine Yonsei University Wonju College of Medicine Wonju 26426 Republic of Korea; ^8^ Division of Natural Product Applied Science University of Science and Technology (UST) Daejeon 34113 Republic of Korea; ^9^ Department of Precision Medicine Graduate School Kyung Hee University Seoul 02447 Republic of Korea; ^10^ Interdisciplinary Program of Genomic Data Science Pusan National University Yangsan 50612 Republic of Korea; ^11^ Department of Anatomy School of Medicine Pusan National University Yangsan 50612 Republic of Korea; ^12^ Department of Biomedical Informatics School of Medicine Pusan National University Yangsan 50612 Republic of Korea; ^13^ Department of Internal Medicine Seoul National University Hospital Seoul 03080 Republic of Korea; ^14^ Department of Biochemistry School of Medicine Pusan National University Yangsan 50612 Republic of Korea; ^15^ Department of Orthopaedic Surgery Pusan National University Hospital and School of Medicine Pusan National University Busan 49241 Republic of Korea; ^16^ Childhood Cancer Research Unit Department of Women's and Children's Health Karolinska Institute Stockholm 17177 Sweden; ^17^ Transplantation Research Center Research Institute for Convergence of Biomedical Science and Technology Pusan National University Yangsan Hospital Yangsan 50612 Republic of Korea; ^18^ Department of Internal Medicine Pusan National University Yangsan Hospital Pusan National University School of Medicine Yangsan 50612 Republic of Korea; ^19^ Institute for Future Earth Pusan National University Busan 46241 Republic of Korea; ^20^ Research Institute for Convergence of Biomedical Science and Technology Pusan National University Yangsan Hospital Yangsan 50612 Republic of Korea

**Keywords:** AML, AP2M1, chemoresistance, HSPC, NOTCH

## Abstract

Acute myeloid leukemia (AML) is a complex hematological malignancy characterized by chemotherapy resistance, leading to poor patient outcomes. This study investigates the role of adaptor protein complex 2 subunit mu 1 (AP2M1) in hematopoiesis and drug response. Utilizing multimodal analyses on bone marrow samples from AML patients and healthy controls, zebrafish models, and human AML cell lines, it is identified that dysregulation of AP2M1 impairs hematopoietic stem and progenitor cell (HSPC) development, underscoring its critical role in hematopoiesis. AP2M1 expression distinctly differentiates normal from malignant cells, surpassing well‐recognized cancer stem cell markers, ATP‐binding cassette transporters, known for drug efflux and chemoresistance. Elevated AP2M1 levels in AML HSPCs correlate with poor clinical outcomes, as its overexpression reduces apoptosis, enhances stemness, and increases drug resistance. In vivo experiments reveal that AP2M1 directly modulates Notch1 expression, amplifying pro‐tumorigenic effects through the Notch1 signaling pathway. These findings highlight the pivotal role of AP2M1 in AML pathogenesis, primarily through its regulation of NOTCH1 expression and signaling cascades. These findings unravel AP2M1 as a previously unrecognized factor in AML pathogenesis and suggest a treatment strategy for AML, focusing on the AP2M1‐NOTCH1 axis.

## Graphical abstract

The precise regulation of AP2M1 is essential for normal hematopoiesis. Overexpression of AP2M1 negatively affects the clinical outcomes of AML patients. Upregulation of AP2M1 enhances stemness and chemoresistance in HSPC cells of AML. AP2M1 modulates NOTCH1 expression, enhancing the Notch1 signaling pathway.

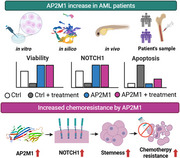

## 1 Introduction

Acute myeloid leukemia (AML) represents a heterogeneous malignancy characterized by the aberrant expansion of clonally derived hematopoietic stem and progenitor cells (HSPCs).^[^ ^1^ ^]^ This neoplastic disorder manifests through the dysregulated proliferation and impaired differentiation of myeloid lineage precursors within the bone marrow microenvironment. Over recent decades, genomic mutations and epigenetic abnormalities have been identified as underlying causes of AML.^[^ ^2^, ^3^ ^]^ For treating AML, Food and Drug Administration (FDA)‐approved targeting and chemotherapeutic agents are being used, which operate through mechanisms such as inhibiting mutant activity, intercalating with DNA to suppress replication and transcription, or inhibiting signaling pathways that promote proliferation and survival.^[^ ^4^, ^5^, ^6^ ^]^ These include FLT3 inhibitors, BCL‐2 inhibitors, CD33‐targeted antibody‐drug conjugates, and anthracycline chemotherapy agents such as midostaurin, venetoclax, gemtuzumab ozogamicin, and idarubicin, respectively.^[^ ^7^, ^8^, ^9^, ^10^ ^]^ While these therapies have shown promise, they face several limitations including drug resistance, limited effectiveness to certain AML subtypes and relapse.^[^ ^7^, ^11^, ^12^ ^]^ Consequently, the outcomes of AML patients are still unsatisfactory. AML patients exhibit a 5‐year overall survival rate of ≈30%, with varying rates by age: ≈50% for younger patients and less than 10% for patients over 60.^[^ ^13^, ^14^ ^]^ This reinforces the urgent need for more effective and targeted therapeutic strategies.

Recently, many researchers have highlighted the crucial role of endocytosis. Dysregulation of endocytic pathways has been implicated in chemotherapy resistance, as it can lead to reduced drug accumulation and increased drug efflux in cancer cells.^[^ ^15^ ^]^ Moreover, endocytosis, a sophisticated cellular mechanism that manages the internalization, packaging, and sorting of cell surface proteins, lipids, and extracellular fluids, plays a significant role in cancer development and progression.^[^ ^15^, ^16^, ^17^ ^]^ One key player in this process is AP2M1, which encodes an adaptor‐related protein complex 2 subunit mu 1 and is responsible for clathrin‐mediated endocytosis (CME) and intracellular trafficking. Previous findings suggested that AP2M1 has a close relationship with various types of cancers. It has been reported that AP2M1 was significantly increased in adenoid cystic carcinoma and mucoepidermoid carcinoma, showing a correlation with the proliferation marker, cyclin D1.^[^ ^18^ ^]^ It was also indicated as a promising prognostic marker in hepatocellular carcinoma.^[^ ^19^ ^]^ In acute lymphoblastic leukemia, AP2M1 has been demonstrated to regulate malignant cell proliferation following treatment with alantolactone.^[^ ^20^ ^]^ Though the role of AP2M1 is poorly established in AML, a study utilizing bioinformatic approaches suggested that AP2M1 could serve as a potential prognostic indicator in AML cases harboring the frequent FLT3‐ITD mutation.^[^ ^21^ ^]^ Despite the proposed possibilities, the functionality of AP2M1 remains largely unexplored not only in blood cancer cells but also in normal blood cell development.

Given the limited understanding of AP2M1, our research aims to elucidate its role by examining its function in normal hematopoiesis and extending its impact on cancer development and drug treatment responses. Since malignant HSPCs, with their self‐renewal capacity, are a primary source of minimal residual disease after treatment and contribute significantly to the high risk of relapse in AML patients,^[^ ^22^, ^23^, ^24^ ^]^ our investigation particularly focuses on the role of AP2M1 in HSPCs. To scrutinize the function of AP2M1 in AML, we conducted multimodal approaches and evaluated its effects on chemoresistance, stem cell‐like properties, and programmed cell death. The effect of AP2M1 was investigated at a cellular resolution using single‐cell RNA sequencing (scRNA‐seq) technology, with validation achieved through in vitro assays, a zebrafish model system, and clinical samples from AML patients. Our results demonstrate that AP2M1 manages these attributes, thereby increasing drug resistance and stemness.

## 2 Results

### 2.1 AP2M1 Plays a Crucial Role in the Emergence of HSPCs

**Figure 1:**
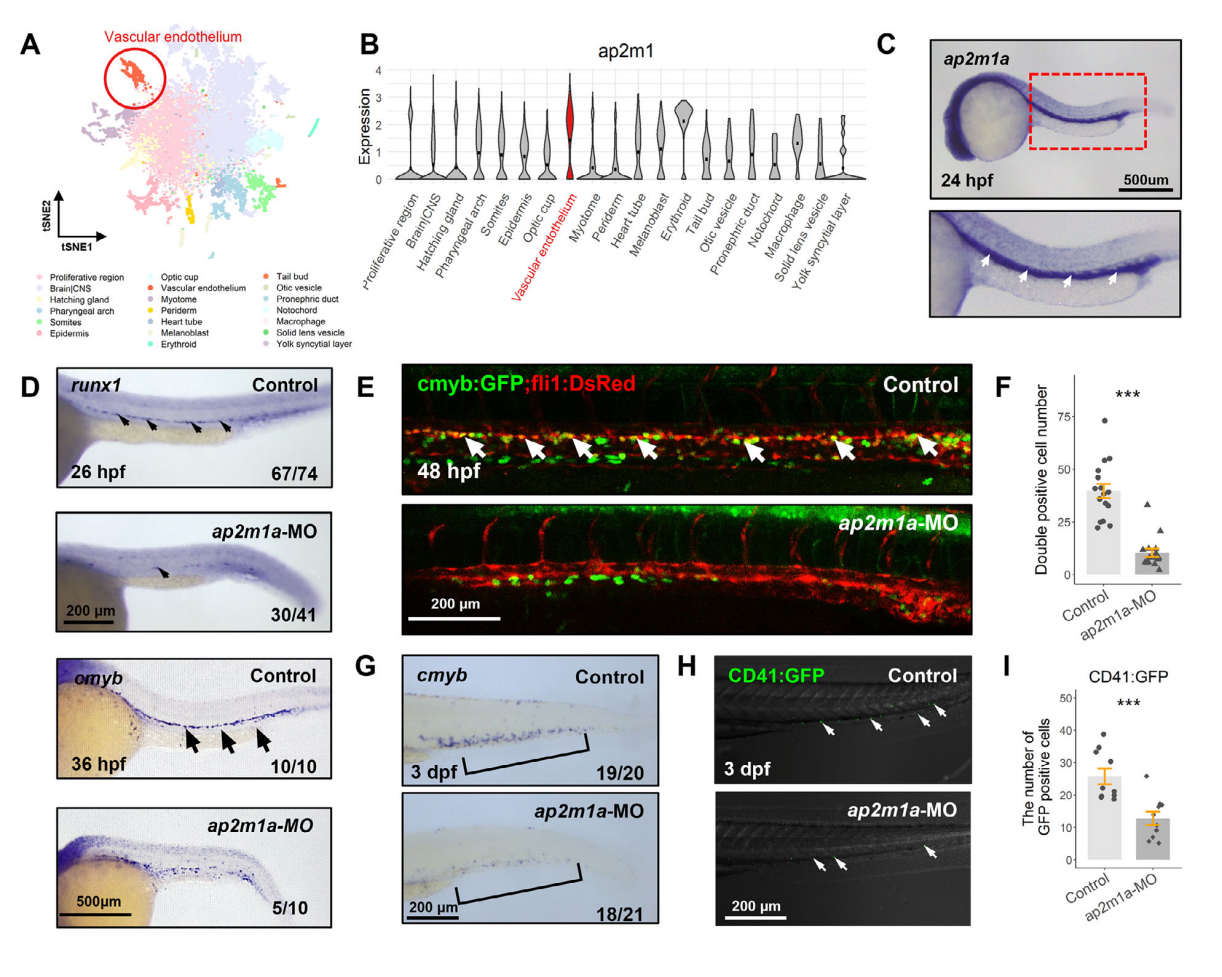
AP2M1a is associated with the emergence of hematopoietic stem cells. A) TSNE plot of scRNA‐seq data derived from normal zebrafish. The color of each dot represents the corresponding cell identity. B) Expression level of *ap2m1* in normal zebrafish. The dots within each violin plot represent the average expression values. C) WISH analysis and lateral view imaging of *ap2m1a* expression pattern in 24 hpf zebrafish embryos. D) WISH analysis and lateral view imaging of *runx1* (26 hpf) and *cmyb* (36 hpf) expression in zebrafish embryos knockdown by injection of *ap2m1a* morpholino (*ap2m1a*‐MO) and un‐injected control zebrafish embryos. E) Fluorescence imaging analysis of cmyb and fli1 expression in the dorsal aorta of 48 hpf zebrafish embryos knocked down by injection of *ap2m1a*‐MO, and in un‐injected control zebrafish embryos. F) Number of double‐positive cells for cmyb and fli1 identified in 48 hpf zebrafish embryos knocked down by injection of *ap2m1a*‐MO, and un‐injected control zebrafish embryos. G) WISH analysis and lateral view imaging of *cmyb* expression in the CHT 3 dpf WT zebrafish embryos knocked down by injection of *ap2m1a*‐MO and un‐injected control zebrafish embryos. Brackets indicate HSPCs at CHT. H) Confocal imaging analysis and lateral view images of CD41 expression in the CHT 3 dpf CD41, GFP zebrafish embryos knocked down by injection of *ap2m1a*‐MO and un‐injected control zebrafish embryos. I) Quantification of CD41‐positive cells in 3 dpf CD41, GFP zebrafish embryos knocked down by injection of *ap2m1a*‐MO and un‐injected control zebrafish embryos.

The overall schematic workflow of this study is illustrated in Figure S1 (Supporting Information). First, to investigate the expression level of *ap2m1a* in each tissue, we explored scRNA‐seq using 24 hpf zebrafish embryos obtained from GSE236393.^[^ ^25^ ^]^ Clustering and annotation were performed on a dataset including 14898 cells derived from normal zebrafish (**Figure** **1**A; Figure S2A, Supporting Information). The expression level of *ap2m1a* was predominantly observed in the vascular endothelium, which is the origin of HSPCs (Figure 1B). WISH results validated a comparable expression pattern of *ap2m1a* in the vascular endothelium (Figure 1C). To inspect the role of *ap2m1a* in the emergence of HSPC, a knockdown experiment was performed by injecting 5.0 ng of MO into zebrafish embryos, which was the necessary dose to decrease the mRNA levels of *ap2m1a* (Figure S2B, Supporting Information). The knockdown of *ap2m1a* reduced the expression of the HSPC‐specific markers *runx1* and *cmyb* in the dorsal aorta (Figure 1D).^[^ ^26^, ^27^ ^]^ Results of WISH were validated using transgenic zebrafish, which specifically mark HSPCs via *cmyb* and *fli1*, following *ap2m1a* knockdown (Figure 1E,F). The reduced HSPCs were not recovered until 3 days postfertilization (dpf) (Figure 1G–I). Thus, these results demonstrate that AP2M1 is an indispensable factor in the development of HSPCs in zebrafish embryos.

### 2.2 AP2M1 is Associated with Poor Clinical Outcomes in AML Patients

**Figure 2:**
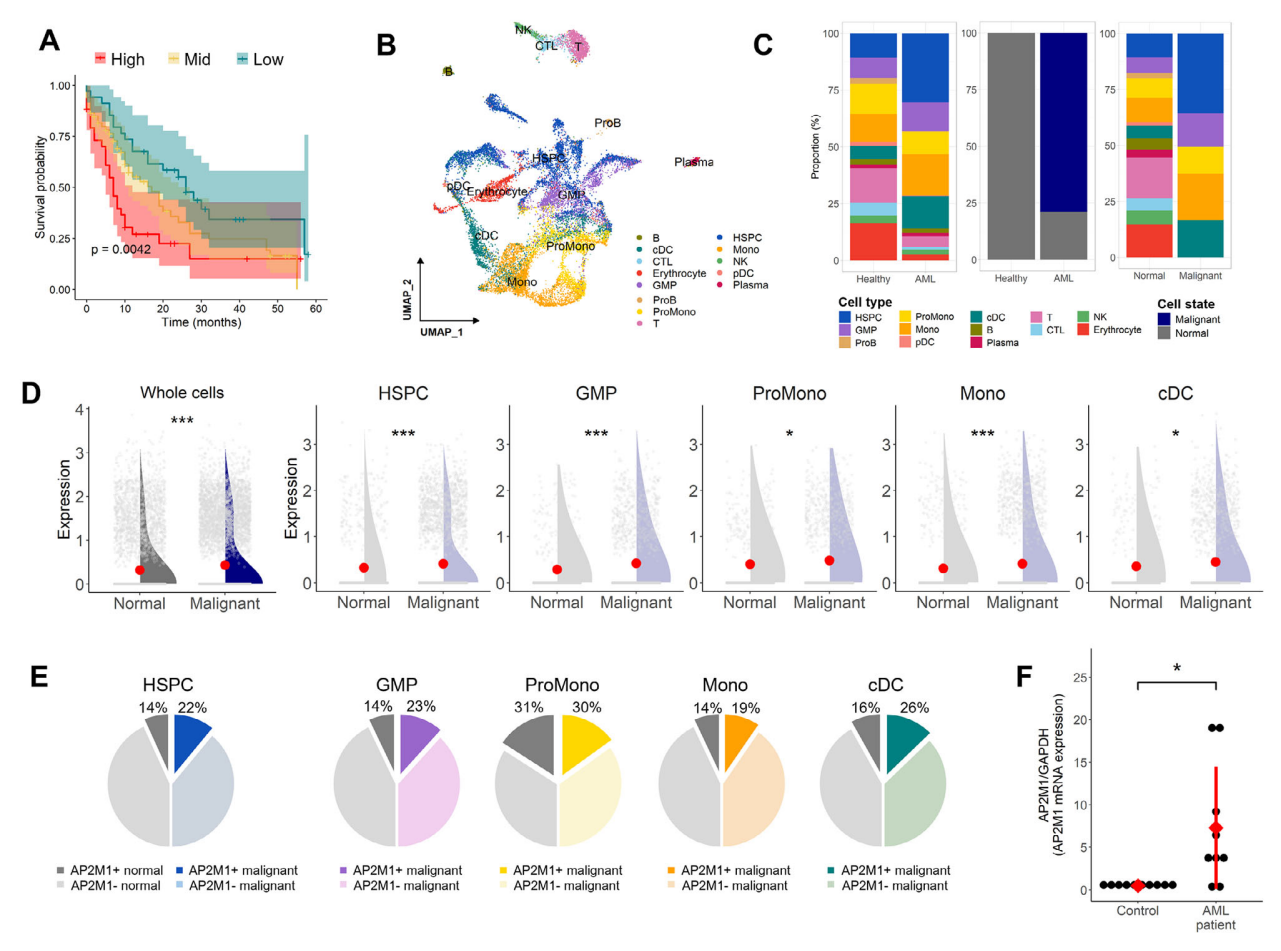
AP2M1 is related to the poor prognosis of AML patients. A) Kaplan‐Meier curve stratified by expression level of *AP2M1* from the TCGA dataset. The low, mid, and high groups included patients with transcriptional levels less than the first quartile, between the first and third quartiles, and higher than the third quartile, respectively. B) Cell types identified on UMAP projections from the bone marrow scRNA‐seq data of AML patients. C) The proportions of cell types and cell states. The first and second bar graphs represent the proportions of cell types and cell states in healthy individuals and AML patients. The third describes the proportions of cell types within each cell state. D) Comparison of *AP2M1* expression levels between normal and malignant cells. The expression levels were compared between all cells and specifically within the five cell types with malignant states, HSPC, GMP, ProMono, Mono, and cDC. ^*^ *p* < .05, ^**^ *p* < 0.01, ^***^ *p* < .001. E) Proportion of *AP2M1*‐expressing cells according to cell states within the five cell types based on scRNA‐seq data. F) The mRNA level of *AP2M1* was quantified in clinical samples. Samples were categorized into two groups, each comprising 10 individuals. qPCR was employed to measure the mRNA levels of *AP2M1*.

To scrutinize the clinical implications of *AP2M1*, we conducted a comparative analysis of survival rates by stratifying 151 patients based on *AP2M1* expression levels. The results indicated that higher expression levels of *AP2M1* are associated with progressively worse prognosis (**Figure** **2**A). To examine the transcriptional level at the cellular resolution, we explored scRNA‐seq data obtained from bone marrow aspirates of AML patients with accession number GSE116256.^[^ ^28^ ^]^ A total of 19418 cells were analyzed, comprising 4670 cells from healthy individuals and 14748 cells from AML patients. The bone marrow aspirate samples included various cell identities, HSPC, granulocyte‐macrophage progenitor (GMP), promonocyte (ProMono), monocyte (Mono), conventional dendritic cell (cDC), plasmacytoid dendritic cell (pDC), progenitor B cell (ProB), B cell, plasma cell, naïve T cell (T), cytotoxic T lymphocyte (CTL), natural killer (NK) cell, and erythrocyte (Figure 2B; Figure S3, Supporting Information). Compared to the bone marrow of healthy individuals, that of AML patients was predominantly composed of HSPCs and GMPs. The majority of cells derived from AML patients showed malignant profiles encompassing HSPC, GMP, ProMono, Mono, and cDC (Figure 2C). The comprehensive transcriptome of AML patients revealed a global upregulation of *AP2M1* expression in malignant cells relative to the normal cells (Figure 2D). In particular, *AP2M1* was upregulated across the entire malignant cellular hierarchy, including HSPCs. The proportion of *AP2M1*+ cells was also expanded in malignant HSPCs (Figure 2E). Similarly, when comparing normal and malignant cells within AML patients, the expression level was also increased in malignant HSPCs (Figure S4A, Supporting Information). We consistently observed raised mRNA levels of *AP2M1* in the qPCR assay of bone marrow samples from AML patients (Figure 2F). We examined the expression of ATP‐binding cassette (ABC) transporters, which, like AP2M1, are responsible for intracellular substance transport and are widely recognized as cancer stem cell markers. Surprisingly, in AML HSPCs, ABC transporters showed remarkably low expression levels and no significant difference in transcriptional level or proportion between normal cells and malignant cells. In contrast, AP2M1 exhibited both increased expression levels and a higher number of expressing cells in malignant HSPCs (Figure S4B,C, Supporting Information). These findings point out the unfavorable impact of aberrant AP2M1 expression in the hematopoietic lineages, specifically HSPCs, and that AP2M1 may play a more prominent role in malignant transformation than previously established markers in AML.

### 2.3 AP2M1 Induces Chemoresistance Through Apoptosis Inhibition in AML

**Figure 3:**
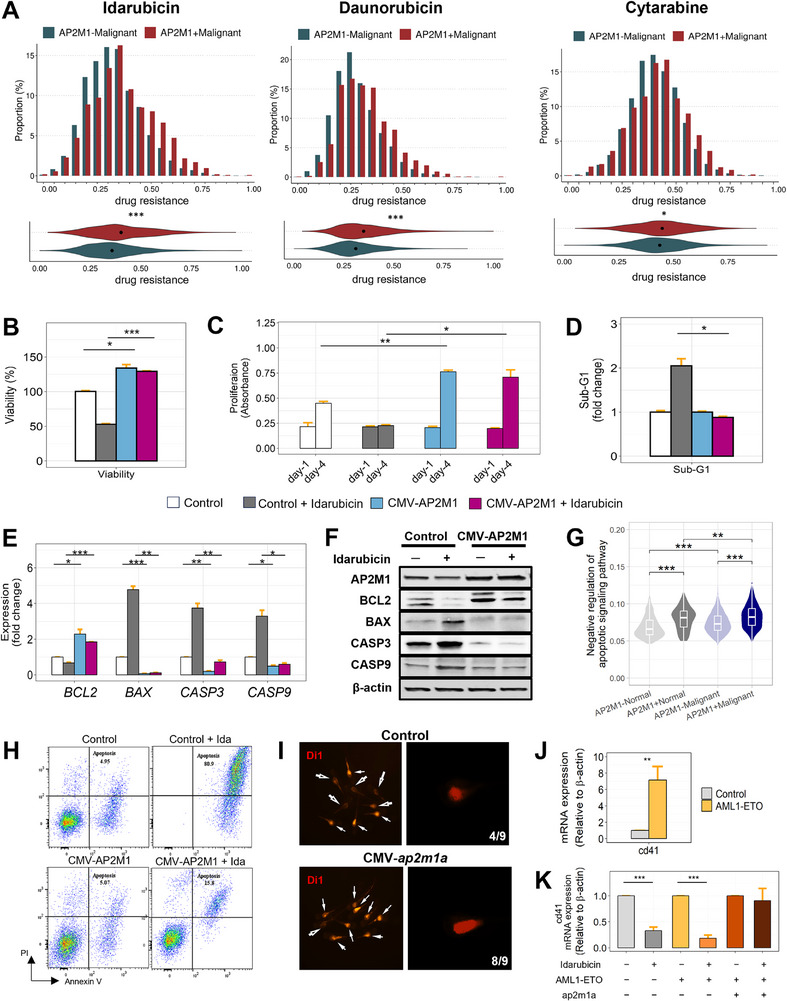
AP2M1 increased drug resistance. A) Putative drug resistance distribution for Idarubicin, Daunorubicin, and Cytarabine in malignant HSPCs of scRNA‐seq data. The upper graph displays the distribution of drug resistance scores, and the lower compares the extent of drug resistance according to *AP2M1* expression. The significance of the difference between groups was compared using the Wilcoxon rank‐sum test. The significance is as follows, ^*^ *p* < .05, ^**^ *p* < 0.01, ^***^ *p* < .001. B,C) Analysis of cell viability (B) and proliferation (C) in AP2M1‐overexpressing cells in response to idarubicin treatment. Control and AP2M1‐overexpressing cells were treated with idarubicin, and cell viability and proliferation were assessed using Cyto X staining. D) Fold change in the sub‐G1 fraction following idarubicin treatment in control and AP2M1‐overexpressing cells. Control and AP2M1‐overexpressing cells were treated with 10 µM idarubicin for 24 h, followed by PI staining. Cell cycle distribution was analyzed by flow cytometry. E,F) Assays evaluating changes in apoptosis‐related genes, including BCL2, BAX, caspase‐3 (CASP3), and caspase‐9 (CASP9). These alterations were assessed in both control and *AP2M1*‐overexpressing cells, as well as following drug treatment, at mRNA (E) and protein levels (F). G) Expression level of genes negatively regulating apoptotic signaling pathway (GO, 2001234). The scores were calculated using transcriptional profiles of HSPC from scRNA‐seq data. H) FACS assay with FITC‐annexin V/PI staining. The x‐axis represents the fluorescence intensity of FITC‐annexin V, indicating the binding of Annexin V to phosphatidylserine on the outer leaflet of the cell membrane, whereas the y‐axis shows the fluorescence intensity of PI, which penetrates cells with compromised membranes and stains their DNA. I) DiI‐stained images in the control and CMV‐*ap2m1a* group. Tumors in the DiI‐stained control group and CMV‐*ap2m1a* group were observed using fluorescence microscopy. Solid arrows indicate the presence of tumors, while hollow arrows denote the disappearance of tumors. J) qPCR analysis of CD41 mRNA expression level in zebrafish embryos injected with AML1‐ETO mRNA at the one‐cell stage and analyzed at 72 hpf. K) qPCR analysis of CD41 mRNA expression level in zebrafish embryos injected at the one‐cell stage with AML1‐ETO mRNA alone or co‐injected with AML1‐ETO and AP2M1 mRNAs. Embryos were treated with idarubicin (10 µM) from 24 to 72 hpf, and CD41 expression was compared between idarubicin‐treated and untreated groups within each injection condition.

Next, we assessed transcriptional status in HSPCs, reflecting drug sensitivity to three key anthracycline‐based chemotherapeutics commonly used in AML treatment, idarubicin, daunorubicin, and cytarabine. The *AP2M1*+ malignant cells demonstrated significantly enhanced drug resistance compared to *AP2M1*‐ malignant cells across all three pharmacological agents (**Figure** **3**A). To investigate the role of *AP2M1* expression in drug resistance in cancer cells, we treated cells with idarubicin and assessed cell viability, proliferation, and sub‐G1 arrest. Cells overexpressing *AP2M1* showed no significant changes in apoptosis after idarubicin treatment, compared to control cells (Figure 3B). The proliferation assays further demonstrated that only control cells, but not *AP2M1*‐overexpressing cells, exhibited proliferation inhibition after idarubicin treatment (Figure 3C). Additionally, cell cycle alterations characterized by sub‐G1 arrest were observed in control cells but not in *AP2M1*‐overexpressing cells (Figure 3D). These results indicate that the expression of *AP2M1* modulates resistance to idarubicin treatment by affecting apoptosis, proliferation, and cell cycle progression in cancer cells. To explore the changes of drug resistance markers at the protein and RNA level, we next performed Western blot and qPCR analyses using BCL2, BAX, Caspase3, and Caspase9. Quantitative assessments were made based on drug dosage and the duration at which the drug's effects were most evident (Figure 3E,F; Figure S5, Supporting Information). BCL2, a negative regulator of the apoptotic process, was increased in the AP2M1‐overexpressing group compared to the control group. Though the level of BCL2 was decreased in the drug‐treated control compared to the untreated control group, the drug‐treated AP2M1‐overexpressing group showed no changes compared to the untreated AP2M1‐overexpressing group. BAX, Caspase3, and Caspase9, which are positive regulators of apoptosis, showed significant increases in the control group following drug treatments. In contrast, although Caspase3 levels showed a variation, the levels of BAX and Caspase9 remained unchanged in the AP2M1‐overexpressing group (Figure 3E,F). In line with these results, the genes involved in the negative regulation of apoptotic signaling were the most strongly expressed in *AP2M1*+ malignant HSPCs compared to normal HSPCs and *AP2M1*‐ malignant HSPCs (Figure 3G). To confirm the apoptosis in response to the drug, we conducted FACS experiments using PI and annexin V and observed changes in apoptosis. The strong apoptotic activity was exclusively detected in the drug‐treated control group. However, upon upregulation of AP2M1, the drug‐induced apoptotic response was substantially diminished by 5 times, suggesting that AP2M1 suppresses drug efficiency (Figure 3H). We next utilized a xenograft model to investigate whether cells with *ap2m1* overexpression exhibit increased resistance to anticancer drugs in vivo (Figure S5C, Supporting Information). In the xenograft model, the AML cell line with *ap2m1* overexpression showed higher drug resistance to idarubicin. We observed that in the control group, four out of nine organisms had remaining cancer cells after anticancer drug treatment, whereas in the *ap2m1* overexpression group, eight out of nine organisms showed residual cancer cells following treatment (Figure 3I).

To evaluate the impact of drug resistance in the zebrafish AML model, we established zebrafish embryo models by injecting AML1‐ETO with and without AP2M1, then analyzed CD41 expression using quantitative PCR. CD41 expression was significantly increased in AML1‐ETO‐injected embryos compared to controls, indicating the expansion of HSCs (Figure 3J). Upon treatment with the chemotherapeutic agent idarubicin, CD41 expression was reduced in both control and AML1‐ETO groups, whereas embryos co‐injected with AML1‐ETO and AP2M1 maintained high levels of CD41 expression after idarubicin treatment (Figure 3K). This multifaceted evidence substantiates the significance of AP2M1 in modulating resistance to chemotherapeutic agents.

### 2.4 AP2M1 Increases the Primitive Nature of Cells in the Bone Marrow

**Figure 4:**
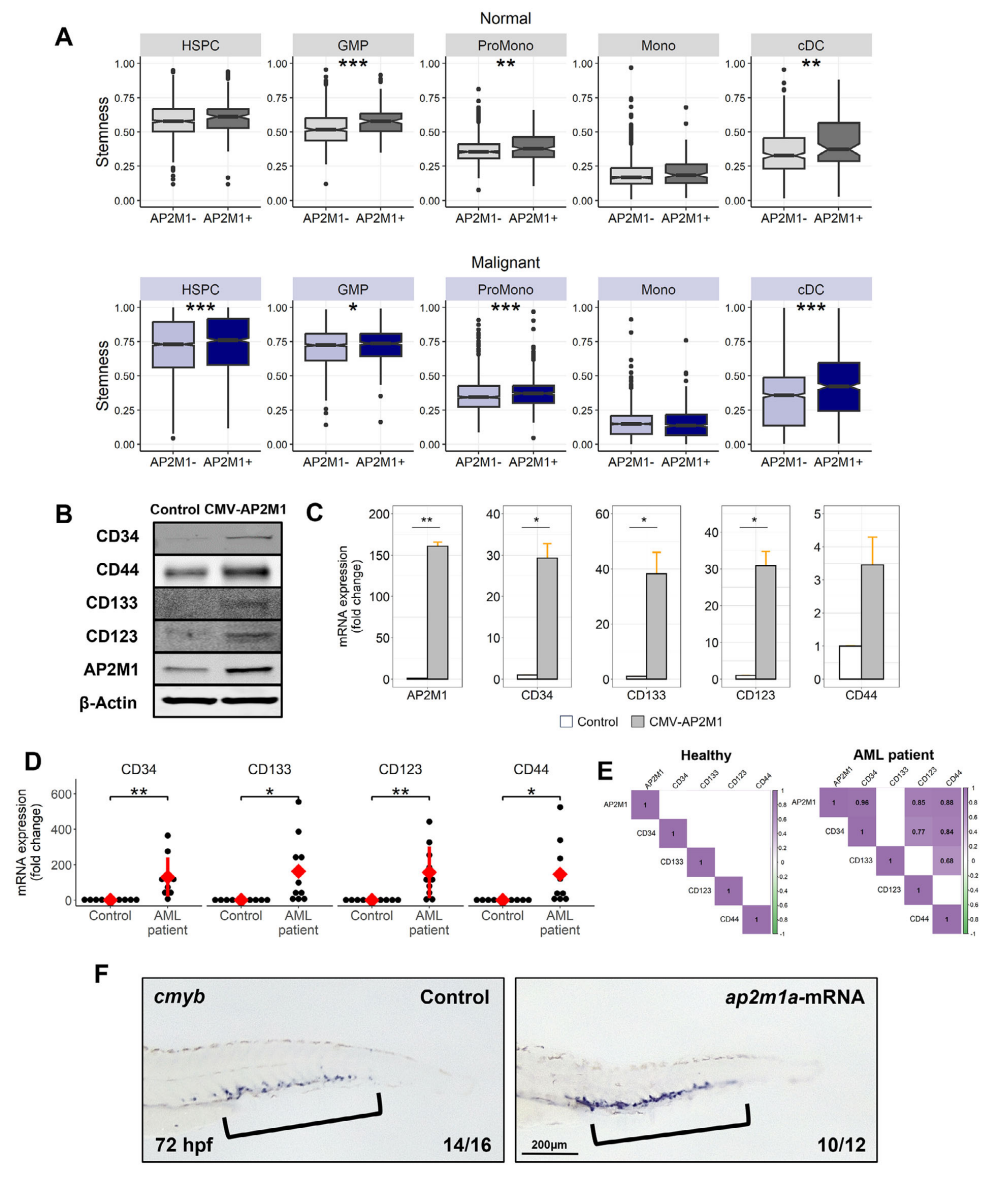
AP2M1 increases stemness. A) CytoTRACE2 stemness score for normal and malignant cell states from scRNA‐seq data. The cells are grouped by the expression of *AP2M1* and by cell compartments. Higher scores suggest greater stem‐like characteristics. The p‐values are as follow, ^*^ *p* < .05, ^**^ *p* < 0.01, ^***^ *p* < .001. B,C) Assays measuring changes in stemness markers. *CD34, CD133, CD123*, and *CD44* were used as indicators of primitive cellular properties. The alterations were assessed in both control and *AP2M1*‐overexpressing cells, as well as following drug treatment, at protein (B) and mRNA levels (C). D) mRNA expression levels of stemness markers in control and AML patient samples. E) Correlation between *AP2M1* and stemness‐related genes in bone marrow samples from healthy individuals and AML patients. Only the significant correlation coefficients are reported. F) WISH analysis and lateral view imaging of *cmyb* expression in the CHT of 72 hpf zebrafish embryos injected with 900 pg nL^−1^ of *ap2m1a* mRNA and WT zebrafish embryos.

Given that self‐renewing and multipotent tumor cells inherently resist anticancer therapies, the observed chemoresistance induced by AP2M1 prompted an investigation into its impact on the stemness in AML patients. Transcriptomic profile analysis demonstrated that stemness was enhanced in *AP2M1*+ malignant cells compared to *AP2M1*‐ malignant cells across most cell compartments, especially within HSPCs. Notably, this difference was not observed between *AP2M1*+ and *AP2M1*‐ normal cells in HSPCs (**Figure** **4**A). Consistent with this observation, only malignant HPSCs demonstrated a positive relationship between *AP2M1* expression and the stemness score among AML patients (Figure S6A, Supporting Information). A consistent elevation of established stemness markers, including CD34, CD133, CD123, and CD44, was observed in the AP2M1‐overexpressing group compared to the control group at both mRNA and protein levels (Figure 4B,C; Figure S6B, Supporting Information). The elevated mRNA level was further confirmed in patient samples (Figure 4D). Moreover, bone marrow samples exhibited a strong co‐expression pattern between *AP2M1* and stemness‐associated genes in AML patients (Figure 4E). Importantly, this correlation was absent in samples from healthy donors. To verify whether overexpression of *ap2m1a* increases stemness similarly to in vitro experiments and patient samples, we injected *ap2m1a* mRNA into zebrafish embryos and examined the *cmyb*, a marker for HSPCs. Through WISH analysis, we confirmed that the number of HSPCs increased in *ap2m1a*‐overexpressing individuals (Figure 4F). These findings collectively suggest that AP2M1 contributes to drug resistance by enhancing the primitive characteristics of cells, potentially through the modulation of stemness‐related pathways.

### 2.5 AP2M1 Activates the Notch Signaling Pathway

**Figure 5:**
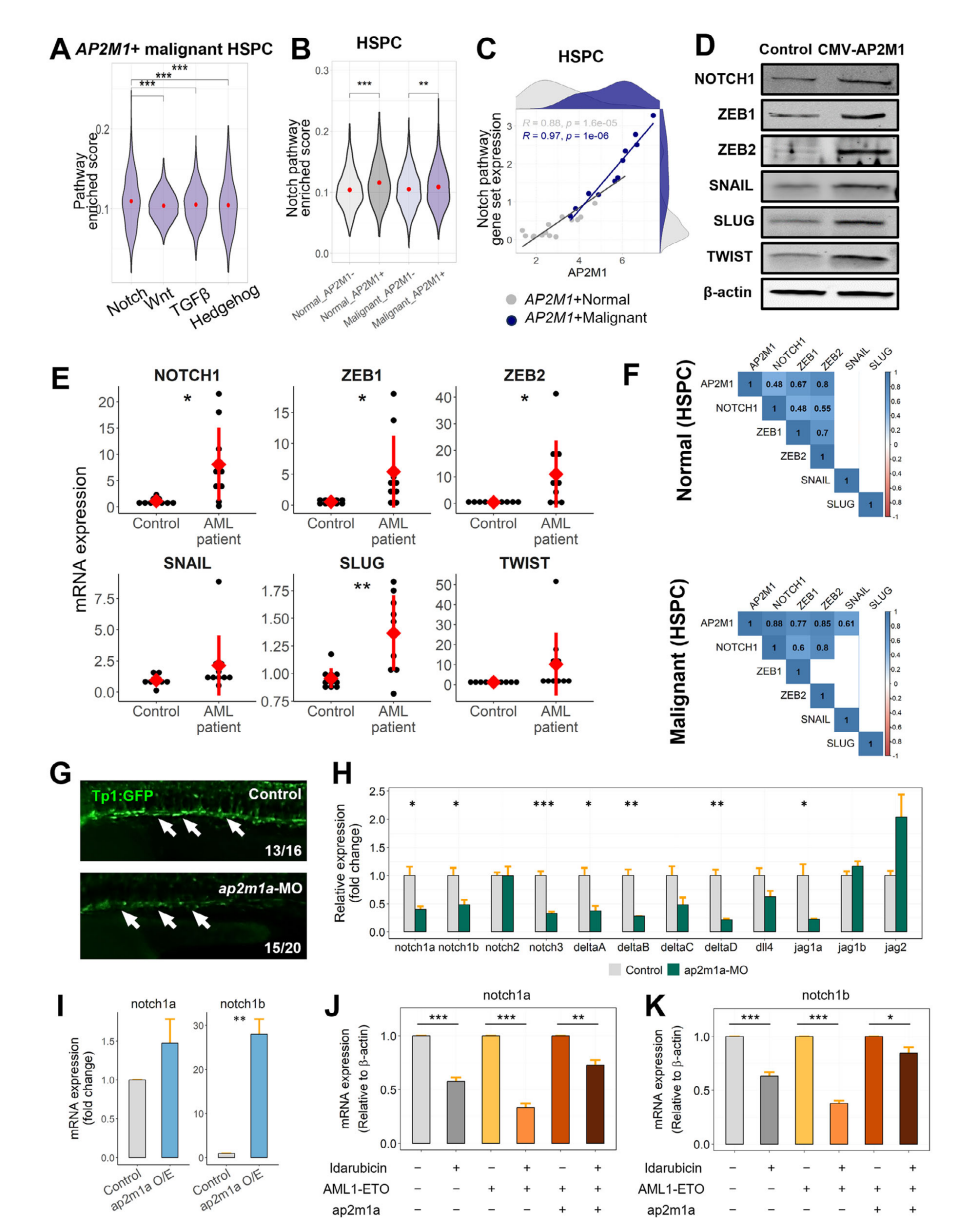
AP2M1 activates the Notch pathway. A) The enrichment score of stemness‐related pathways, Notch, Wnt, TGFβ, and Hedgehog signaling pathways in *AP2M1*+ malignant HSPCs from scRNA‐seq data. The significance is as follows, ^*^ *p* < .05, ^**^ *p* < 0.01, ^***^ *p* < .001. B) The enrichment score of the Notch signaling pathway according to cell malignancy and *AP2M1* expression in HSPCs from scRNA‐seq data. C) The correlation between *AP2M1* expression and the Notch signaling gene set expression in HSPCs from scRNA‐seq data. The density plots above the horizontal and vertical axes illustrate the distribution of *AP2M1* and Notch pathway gene set expression, respectively. The correlation coefficients and p‐values are described at the top left. D) The protein levels of genes involved in the Notch1 signaling pathway. The proteins are assessed in both control and *AP2M1*‐overexpressing cell lines. E) The mRNA expression levels of Notch1 pathway genes in control and AML patient samples. F) Correlation between *AP2M1* and Notch pathway genes in normal and malignant HSPCs from scRNA‐seq dataset. Only the significant correlation coefficients are shown. G) Fluorescence imaging analysis of *Tp1* expression in Tp1, GFP zebrafish embryos. The knockdown zebrafish embryos by injection of *ap2m1a*‐MO and un‐injected control zebrafish embryos are shown. H) qPCR analysis of relative expression of Notch signaling pathway genes in zebrafish embryos with *ap2m1a*‐MO knockdown. I) mRNA expression levels of *notch1a* and *notch1b* in response to *ap2m1a* overexpression. 900 pg nL^−1^ of *ap2m1a* mRNA was injected into zebrafish embryos. J,K) qPCR analysis of *notch1a* and *notch1b* mRNA expression level in zebrafish embryos injected at the one‐cell stage with AML1‐ETO mRNA alone or co‐injected with AML1‐ETO and AP2M1 mRNAs. Embryos were treated with idarubicin (10 nm) from 24 to 72 hpf, and *notch1a* and *notch1b* mRNA expression was compared between idarubicin‐treated and untreated groups within each injection condition.

To elucidate how AP2M1 enhances stemness in AML, we first profiled the intensity of signaling pathways known to regulate self‐renewal, proliferation, and apoptosis of HSPCs, including Notch, Wnt, TGFβ, and Hedgehog.^[^ ^29^, ^30^, ^31^ ^]^ Among these pathways, Notch signaling was notably the most enriched in *AP2M1*+ malignant HSPCs (**Figure** **5**A). *AP2M1*+ HSPCs from both normal and malignant cells displayed higher Notch signaling enrichment scores compared to their *AP2M1*‐ counterparts (Figure 5B; Figure S7A, Supporting Information). Genes involved in the Notch signaling pathway exhibited not only higher transcriptional distribution but also a more pronounced correlation with *AP2M1* expression in malignant HSPCs compared to normal HSPCs (Figure 5C). We investigated the downstream genes of the Notch pathway, including ZEB1, ZEB2, SNAIL, SLUG, and TWIST. In cells with increased AP2M1 expression, there was upregulation of the Notch pathway target genes (Figure 5D). Furthermore, we confirmed elevated transcriptional levels of the genes in AML patients (Figure 5E; Figure S7D,E, Supporting Information). Consistent with these observations, *AP2M1* showed higher co‐expression with Notch pathway genes in malignant HSPCs compared to normal HSPCs, further supporting the relationship between *AP2M1* and Notch signaling in AML (Figure 5F). Next, following *ap2m1* knockdown in Tp1, GFP transgenic zebrafish embryos, we employed fluorescence microscopy to visualize the effects on Notch signaling activity. The TP1 fluorescence expression was reduced in the *ap2m1* knockdown group compared to the control group (Figure 5G). Moreover, qPCR analysis of *ap2m1* knockdown zebrafish embryos revealed an overall reduction in the expression of Notch signaling pathway genes (Figure 5H). Specifically, a significant reduction in the expression of *notch1b*, *notch3, deltaA, deltaB, deltaD, jag1a*, and *jag1b* was observed. We further examined the effects on notch1 signaling by overexpressing *ap2m1a* in zebrafish embryos through mRNA injection. The results showed that overexpression of ap2m1a via mRNA led to an increasing tendency in notch1a expression, while notch1b expression exhibited a nearly 30‐fold increase (Figure 5I). To explore whether the alternative endocytic pathways or related factors contribute to the regulation of Notch signaling, we assessed the roles of clathrin, endophilin, and caveolin. Initially, we first measured the mRNA expression levels of clathrin and endophilin in AP2M1‐overexpressing cells, observing that clathrin expression was affected by AP2M1 regulation (Figure S7B, Supporting Information). To further elucidate their roles in Notch signaling modulation, we overexpressed endophilin and clathrin, respectively, and examined the transcriptional changes in the Notch target genes. Although overexpression of endophilin and clathrin led to elevated expression levels, the effects on Notch target gene transcription were substantially less pronounced than those seen with AP2M1 overexpression (Figure 3E,I; Figure S7C, Supporting Information). Additionally, caveolin expression did not align with a positive regulation of Notch pathway‐related gene expression (Figure S7D,E, Supporting Information). Therefore, our findings collectively identify AP2M1 as a specific and key effector regulating the Notch signaling pathway.

To further validate the regulatory role of AP2M1 in the Notch signaling pathway in vivo, we analyzed *notch1a* and *notch1b* expression in zebrafish AML models. Under idarubicin treatment, *notch1a* and *notch1b* expression levels were significantly reduced in both control and AML1‐ETO‐only groups. In contrast, embryos co‐injected with AML1‐ETO and ap2m1a mRNA maintained relatively high expression levels of *notch1a* and *notch1b* (Figure 5J,K). These results indicate that modulation of *AP2M1* levels leads to consequential changes in *NOTCH1* level and the Notch1 signaling pathway, suggesting a regulatory role for *AP2M1* in the Notch pathway cascade.

## 3 Discussion

Our research has newly elucidated the crucial role of *ap2m1a*, which is a zebrafish ortholog of human AP2M1, in HSCP development. We discovered that *ap2m1a* is highly expressed in the vascular endothelium, where HSPCs emerge, in zebrafish embryos. We thus investigated the role of *ap2m1a* at 3 dpf, corresponding to the definitive wave, which is the developing process of HSPCs.^[^ ^32^, ^33^ ^]^ The knockdown of *ap2m1a* reduced the number of *cmyb* and *fli1* double‐positive cells in the dorsal aorta (Figure 1E). Additionally, other HSPCs markers, *runx1* and *CD41*, were also decreased in *ap2m1a* morphants (Figure 1D,H). These observations indicate that *ap2m1a* is closely involved in the differentiation of normal HSPCs. Therefore, dysregulation of *ap2m1a* could disrupt the balance of normal hematopoiesis.

Our study employed a multifaceted approach, integrating in vitro, in vivo, and in silico methodologies, to elucidate the consequences of changes in *AP2M1* expression. The findings revealed that AP2M1 plays a pivotal role in modulating apoptosis and stemness, thereby contributing to drug resistance in HSPCs of AML. We confirmed these cancer‐favorable properties are closely associated with the Notch1 signaling pathway, which is most prominently activated in malignant *AP2M1*+ HSPCs of human samples. Importantly, our findings revealed that *AP2M1* exerts a significant influence on the expression levels of *NOTCH1*, a key regulatory gene in hematopoiesis and cancer development. Overexpression of *AP2M1* in HL60 cells resulted in increased levels of *NOTCH1* and its downstream genes, whereas knockdown of *ap2m1* in zebrafish led to reduced expression of *notch1*. Additionally, *AP2M1* was considerably correlated with *NOTCH1* and its target genes in the malignant HSPC compartment. To investigate the potential cross‐talk between Notch1 expression and other regulatory pathways, we compared the impact of clathrin, endophilin, and caveolin. AP2M1 has been recognized as an adaptor protein in CME by binding to specific motifs within the cytoplasmic domains of cargo proteins, recruiting clathrin to the plasma membrane, and initiating the formation of clathrin‐coated pits. In contrast, endophilin induces membrane curvature, facilitating the formation and constriction of vesicle necks, and contributing to vesicle scission and uncoating.^[^ ^34^ ^]^ Recent studies have identified endophilin as a key regulator not only in CME but also in clathrin‐independent endocytosis (CIE), termed fast endophilin‐mediated endocytosis.^[^ ^35^, ^36^ ^]^  The caveolin‐mediated pathway is considered one of the major forms of CIE, important for the uptake of lipids, certain toxins, and signal transduction.^[^ ^37^ ^]^ Our results demonstrated that increased AP2M1 expression elevates clathrin without affecting endophilin levels, and that modulation of AP2M1 produces a more pronounced impact on the expression of Notch1 signaling pathway genes than the individual regulation of endophilin, clathrin, or caveolin. These findings suggest that AP2M1 plays a primary role in endocytosis for regulating Notch1 expression. In addition, consequently, a relative increase in CME may lead to a higher proportion of internalization of cargoes specific to this pathway and trigger sequential changes in interconnected endocytic pathways.

One possible mechanism for increasing the expression of Notch1 target genes may involve the nuclear factor (NF)‐κB pathway. Notch intracellular domain produced through clathrin‐mediated endocytosis can migrate into the nucleus or remain in the cytoplasm, where it interacts with other signaling pathways, including NF‐κB, mTORC2, AKT, and Wnt.^[^ ^38^, ^39^ ^]^ In T cells, NF‐κB subunits containing RelA have been reported to bind to the *NOTCH1* promoter region, thereby upregulating Notch1 expression.^[^ ^40^ ^]^ However, due to the context‐dependent nature of *NOTCH1* function and regulation, further investigation into the interplay between Notch1 and NF‐κB signaling in HSPCs is necessary.

Currently, a variety of approaches are being attempted for the treatment of AML. Stem cell transplantation, anthracycline drugs such as idarubicin or daunorubicin, and targeted therapies like midostaurin, ivosidenib, or enasidenib are being utilized.^[^ ^41^, ^42^, ^43^, ^44^, ^45^, ^46^ ^]^ However, these treatments are limited by high risks of complications, including graft‐versus‐host disease, toxic side effects, resistance, and effectiveness confined to specific molecular subtypes.^[^ ^41^, ^47^, ^48^, ^49^, ^50^ ^]^ In response to the necessity for additional therapeutic agents, our results suggest *AP2M1* as a clinically applicable candidate in AML. Based on findings obtained from human bone marrow specimens, *AP2M1* showed substantially high expression in AML patients, while its expression in healthy bone marrow was notably low. *AP2M1* expression in AML specimens was 19‐fold higher on average compared to normal specimens. Moreover, *AP2M1* showed a strong correlation with primitive indicators, such as *CD34*, *CD123*, and *CD44* in the HSPCs of AML patients (Figure 4E). In contrast, normal HSPCs showed no significant relationships and markedly lower expression. Taken together, these findings indicate that *AP2M1* could be useful as an AML‐specific therapeutic target, potentially offering an alternative option for patients who are challenging to treat with existing therapies.

One potential application of our findings is the utilization of AP2M1 expression levels as a determinant for idarubicin administration in treatment protocols. AP2M1 overexpression demonstrated increased drug resistance in both AML cell lines and xenograft zebrafish models. Furthermore, the intensity of drug resistance inferred from HSPC profiles in AML patient scRNA‐seq data corroborated these findings, showing a positive association between *AP2M1* expression and enhanced resistance. The worsening of overall survival rates sequentially correlated with *AP2M1* expression levels. This, along with consistent results across multiple experimental platforms, strongly supports the potential of AP2M1 as a biomarker for guiding pharmacological interventions in AML treatment. We believe that future studies, such as experiments examining changes in drug resistance after overexpressing AP2M1 in bone marrow specimens, would yield more definitive data.

In conclusion, this research provides new insights into the molecular mechanisms by which AP2M1 influences malignant hematopoietic development in AML. Our study suggests that the upregulation of AP2M1 generates a positive feedback loop within the Notch1 signaling pathway, serving as a key regulatory node that enhances stemness and drug resistance.

## 4 Experimental Section

### Human Specimen Collection

A bone marrow specimen was obtained from ten patients with acute myeloid leukemia and ten patients who underwent bone marrow examination but were not diagnosed with hematologic disease. Extra bone marrow specimens were collected and processed in accordance with the institute's protocol during the diagnostic bone marrow examination. Bone marrow specimens were stored at the Samsung Medical Center Biobank after the following preparation process.

### Human Sample Preparation

A bone marrow specimen was collected in an EDTA tube. After adding 40 mL of RBC lysis buffer to a 50 mL conical tube, 10 mL of the whole bone marrow sample was added to the conical tube. After incubation for 5 min at room temperature, the tube was centrifuged at 3000 RPM at 4 °C for 3 min. The supernatant was discarded, and 20mL RBC lysis buffer was added. After mixing, the specimens were centrifuged at 3000 RPM at 4 °C for 3 min. After discarding the supernatant, 20 mL of PBS was added, and were mixed was then centrifuged at 3000 RPM at 4 °C for 3 min. After discarding the supernatant, 2.5 mL of Cellbanker (Amsbio) was added and mixed using a pipette. The specimen was dispensed 0.5mL each into a cryotube and stored in the refrigerator at −80 °C.

### Maintenance of Zebrafish

Wild‐type zebrafish AB and transgenic zebrafish (*cmyb*, GFP, fli1, DsRed, and CD41, GFP) were maintained automatic circulation aquarium system (Techniplast, Maggio, Italy) at 28.5 °C with 14h light/ 10h dark cycle (pH, 7.0 electrical conductivity, 1000 microsiemens) following guide for IACUC (Institutional Animal Care and Use Committee) from Pusan National University (PNU‐2023‐0359). Zebrafish embryos were maintained in an incubator at 28 ± 1 °C with E3 solution (14.61 g NaCl, 0.63 g KCl, 1.83 g CaCl_2_·2H_2_O, and 1.99 g MgSO_4_ in 1 L of deionized water). All transgenic zebrafish were kindly supported by the Institute of Basic Science, Center for Genomic Integrity (IBS‐CGI, Ulsan, Korea).

### Microinjection of Morpholino (MO)

A splicing‐blocking morpholino targeting *ap2m1a* (Gene Tools, Philomath, USA) was resolved in DEPC water in 25 ng nL^−1^ stock. The sequence of *ap2m1a*‐MO was 5′‐ GGAAACAAACGTCTTACCATCCAGC ‐3′. The morpholino targeting *ap2m1a* was injected into embryos of wild‐type AB zebrafish or transgenic zebrafish embryos at the one‐cell stage of development. Microinjections were performed with a Femtojet 4i microinjector (Eppendorf, Hamburg, Germany).

### RNA Isolation and RT‐PCR using Zebrafish Embryo

To isolate, zebrafish embryos were sacrificed using tricaine. Then, sacrificed zebrafish embryos were homogenized using a pestle. Total RNA was isolated from homogenised zebrafish embryos using 1 mL TRIzol reagent (Molecular Research Center Inc., Cincinnati, USA). Chloroform was added and vortexed for protein separation, and isopropanol was added for precipitation of total RNA. Three micrograms of total RNA were reverse transcribed using SuperScript IV Reverse Transcriptase (Thermo Fisher, Waltham, Massachusetts, MA, USA). RT‐PCR was performed using GoTaq G2 DNA Polymerase (Promega, Madison, Wisconsin, USA) and visualised using the BANDi‐Green Nucleic Acid Stain (Translab, Daejeon, Korea).

### Whole‐Mount In Situ Hybridization (WISH)

WISH was performed according to the previous methods.^[^ ^51^ ^]^ Briefly described, embryos were fixed using 4% paraformaldehyde in phosphate‐buffered saline (PBS) and dehydrated with methanol at −20 °C overnight for permeabilization. Samples were incubated with cold acetone for permeabilization. Then, samples were hybridised with a digoxigenin (DIG)‐labelled antisense RNA probe in hybridisation buffer (50% formamide, 5× SSC, 500 µg mL^−1^ Torula yeast tRNA, 50 µg mL^−1^ heparin, 0.1% Tween‐20, and 9 mm citric acid (pH 6.5)) for 3 days. The samples were washed using 0.2× SSC solutions. Washed samples were blocked with blocking buffer (normal goat serum and bovine serum albumin), and next incubated with alkaline phosphate‐conjugated DIG antibodies (1, 4000) (Roche, Mannheim, Germany) overnight at 4 °C. Samples were incubated with alkaline phosphatase reaction buffer (100 mm Tris (pH 9.5), 50 mm MgCl_2_, 100 mm NaCl and 0.1% Tween‐20) and the NBT/BCIP substrate (Promega, Madison, Wisconsin, USA) for visualisation of the WISH signal.

### AML Model using Zebrafish Larvae

The human pCMV‐AML1‐ETO fusion gene (plasmid #12 428, Addgene Watertown, MA, USA) was subcloned into the pCS2+ expression vector for mRNA synthesis. The insert and vector were digested with appropriate restriction enzymes and ligated using NEB T4 DNA ligase (Cat# M0202, New England Biolabs, Ipswich, MA, USA) according to the manufacturer's protocol. The ligation product was transformed into Escherichia coli, and mRNA was synthesized using the mMESSAGE mMACHINE SP6 kit (Cat# AM1340, Thermo Fisher Scientific, Waltham, MA, USA). The synthesized mRNA was diluted to a concentration of 150 pg nL^−1^ and microinjected into single‐cell stage zebrafish embryos.

### Xenograft Model using Zebrafish Larvae

Control cells and CMV‐AP2M1 stable cells were centrifuged and resuspended in 1 mL of RPMI (Thermo Fisher, Waltham, Massachusetts, MA, USA). Following resuspension, 4 µL of DiI was added, and the cells were incubated for 10 min at 37 °C, then placed on ice under dark conditions for 15 min. Afterward, 4 µL of 500 mm EDTA was added, and the cells were centrifuged to complete the staining process. The concentration of stained cells was determined to be 1.25 × 10^8^ cells mL^−1^ using a hemocytometer. One hundred twenty‐five DiI‐stained cells were microinjected into the yolk of zebrafish embryos at 48 h post fertilization (hpf). Six hours post‐injection, the zebrafish embryos were treated with 10 µm Idarubicin in E3‐PTU media. After 24 h of incubation, tumor formation was observed using a fluorescence microscope (Discovery V8, Carl Zeiss, Oberkochen, Germany).″

### Confocal Microscope Imaging

Transgenic zebrafish embryos were anesthetized with tricaine. Then, anesthetized embryos were mounted at glass‐bottom dish and observed with a confocal microscope (LSM880, Carl Zeiss, Oberkochen, Germany).

### Chemical Reagents and Antibodies

Antibodies against AP2M1, Bcl2, Bax, Caspase‐3, Caspase‐9, CD34, CD44, CD113, CD123, Notch1, Zeb1, Zeb2, snail, Slug, Twist, and β‐actin were purchased from abclonal (Woburn, MA, USA). IRDye‐conjugated IgG antibody (925‐68070, 925–32211, Li‐COR Bioscience, Lincoln, NE, USA) was used as the secondary antibody. All other reagents, including idarubicin, were obtained from Sigma–Aldrich (St Louis, MO, USA), unless otherwise indicated.

### Cell Culture and Transfection

HL60 cells (KCLB No. 10240, RRID,  CVCL_0002) were obtained from the Korean Cell Line Bank in 2023. Although mycoplasma contamination was not directly tested in the lab, the cells were treated with Mycoplasma Removal Agent (MP Biomedicals, Santa Ana, CA, USA) at the recommended concentration of 0.5 µg mL^−1^ prior to use. The HL60 cells were cultured in RPMI‑1640 medium (Gibco BRL, Grand Island, NY, USA), supplemented with 10% fetal bovine serum and 1% antibiotic‑antimycotic solution (Gibco BRL). Cells were incubated at 37 °C in a humidified atmosphere containing 5% CO_2_. For overexpression of target proteins, pCMV3‐C‐Myc‐AP2M1 (HG16144‐CM) and pCMV3‐clathrin (HG14036‐CF) were purchased from Sinobiological (South Korea), and pcDNA3‐endophilin (#47 403) was obtained from Addgene (Watertown, MA, USA). Cells were transfected with a control vector for 12 h using Lipofectamine 2000 (Invitrogen, Waltham, MA, USA), according to the manufacturer's protocol.

### Western Blotting

For western blot assays, cells were lysed with essentially the same buffer and mixed with the sample buffer. Samples were subjected to 10% polyacrylamide gel electrophoresis and transferred onto a nitrocellulose membrane. Membranes were incubated with the indicated primary antibodies and IRDye‐conjugated secondary antibodies (Li‐COR Biosciences, Lincoln, NE, USA), and protein bands were visualized using an Infrared image analyzer (Li‐COR Biosciences).

### Quantitative Real‐Time Polymerase Chain Reaction (qPCR)

Total RNA was extracted using a RNeasy Mini Kit (Qiagen). Complementary DNA (cDNA) was synthesized using the Smart Gene Compact cDNA Synthesis Kit (Smart Gene, South Korea). Primers targeting human AP2M1 were 5′‐atcgtcggaatgagctcttcct‐3′ (forward) and 5′‐ gtcccacttctcgcactagc‐3′ (reverse), human Notch1 were 5′‐actgtgaggacctggtggac‐3′ (forward) and 5′‐ttgtaggtgttggggaggtc‐3′ (reverse), human Zeb1 were 5′‐ggcatacacctactcaactacgg‐3′ (forward) and 5′‐tgggcggtgtagaatcagagtc‐3′ (reverse), human Zeb2 were 5′‐cgcttgacatcactgaagga‐3′ (forward) and 5′‐cttgccacactctgtgcatt‐3′ (reverse), human Snail were 5′‐tttaccttccagcagcccta‐3′ (forward) and 5′‐cccactgtcctcatctgaca‐3′ (reverse), human Slug were 5′‐ctttttcttgccctcactgc‐3′ (forward) and 5′‐acagcagccagattcctcat‐3′ (reverse), human Twist were 5′‐gtccgcagtcttacgaggag‐3′ (forward) and 5′‐ccagcttgagggtctgaatc‐3′ (reverse), human CD34 were 5′‐caccctgtgtctcaacatgg‐3′ (forward) and 5′‐ggcttcaaggttgtctctgg‐3′ (reverse), human CD44 were 5′‐agcaaccaagaggcaagaaa‐3′ (forward) and 5′‐gtgtggttgaaatggtgctg‐3′ (reverse), human CD123 were 5′‐cccaacatgactgcaaagtg‐3′ (forward) and 5′‐tctttcccgggctcttattt‐3′ (reverse), human CD133 were 5′‐ttgtggcaaatcaccaggta‐3′ (forward) and 5′‐tcagatctgtgaacgccttg‐3′ (reverse), human endophilin were 5′‐tgattgcaactttggcccag‐3′ (forward) and 5′‐atcaaaatccaggcgtcgac‐3′ (reverse), human clathrin were 5′‐aaagcccagttgcagaaagg‐3′ (forward) and 5′‐actctcgagccttcttacgg‐3′ (reverse) and those for human GAPDH were 5′‐ccaatgtgtccgtcgtggatc‐3′ (forward) and 5′‐gttgaagtcgcaggagacaac‐3′ (reverse). qPCR was performed using a LightCycler 96 Real‐Time PCR System (Roche, Basel, Switzerland). Target mRNA expression relative to the housekeeping gene expression (GAPDH) was calculated using the ΔΔCT method.

### Fluorescence‐Activated Cell Sorting (FACS) Analysis

Apoptosis and cell cycle progression were analyzed using flow cytometry (FACS) in HL60 cells. To assess apoptosis, cells were stained using the Annexin V‐FITC/PI Apoptosis Detection Kit (Sigma–Aldrich) according to the manufacturer's instructions. For cell cycle analysis, cells were harvested, washed twice with cold PBS, and fixed in 70% ethanol at 4 °C overnight. After fixation, cells were treated with RNase A (100 µg mL^−1^) at 37 °C for 30 min, followed by staining with Propidium Iodide (PI, 50 µg mL^−1^) for 30 min at room temperature in the dark. DNA content was then analyzed using a flow cytometer (BD FACS Canto II, BD Biosciences, San Jose, CA, USA), and the distribution of cells across the G0/G1, S, and G2/M phases was determined using BD FACSDiva software.

### Cell Viability and Proliferation Assay

Cell viability and proliferation were assessed using the Cyto X (LPS solution, Korea). For the viability assay, cells were seeded in 96‐well plates at a density of 1 × 10⁴ cells per well and treated with idarubicin for 24 h. For the proliferation assay, cells were treated under the same conditions and incubated for 4 days. After the respective incubation periods, 10 µL of Cyto X reagent was added to each well, followed by incubation at 37 °C for 2 h. The absorbance was measured at 450 nm using a microplate reader (Allsheng, AMR‐100, China). The results were expressed as relative viability and proliferation compared to the untreated control group. All experiments were performed in triplicate.

### Processing and Survival Analysis of TCGA Dataset

Gene expression quantification released data (39.0) from the TCGA‐LAML project were gathered using TCGAbiolinks.^[^ ^52^ ^]^ The data consisted of transcripts per million counts for gene expression levels. Survival months and follow‐up durations were collected, excluding patients missing either metric. The Kaplan‐Meier survival analysis and log‐rank test were conducted using survival (v3.4‐0) and survminer (v0.4.9).^[^ ^53^ ^]^

### Preprocessing scRNA‐seq Dataset of AML Patients

The single‐cell RNA profiles of human bone marrow aspirates, along with information on cell malignancy and annotated cell type, were downloaded from the Gene Expression Omnibus (GEO) database under accession number GSE116256.^[^ ^28^ ^]^ Samples collected at the time of diagnosis before chemotherapy were selected, and a sample that underwent a different storage process was excluded. The scRNA‐seq data were loaded and processed on an R (v4.3.2) environment and Seurat packages (v4.4.0).^[^ ^54^ ^]^ The cells remained according to the following criteria: more than 1000 transcripts, 500 expressed genes, and less than 20% mitochondrial RNA percentage. The samples with fewer than 50 cells were excluded from downstream processing. The read counts were normalized and scaled, also the variable features were identified within each sample using the NormalizeData, ScaleData, and FindVariableFeatures functions. Then, the integration features were selected using the SelectIntegrationFeatures function. The reciprocal principal component (PC) analysis method was employed, and the largest sample among healthy donors was used as a reference in the integration step. To assess cellular malignancy, primers targeting sequences adjacent to mutations and a supervised random forest classifier were employed. For training the random forest classifier, transcriptome data from normal cells obtained from healthy donors and malignant cells harboring detected mutations were used as training data. To identify mutations, the authors of a previous study performed targeted DNA sequencing on samples from AML patients using a gene panel prior to single‐cell sequencing, allowing for the identification of key mutations.^[^ ^28^ ^]^ Based on the selected mutation sites, a total of 43 primers were designed. Short‐read sequencing was then conducted using primers specifically designed to amplify only cDNA fragments containing the pre‐identified mutation sites, utilizing a subset of the whole transcriptome amplification cDNA products generated from scRNA‐seq. Cells in which mutated sequences were detected were classified as malignant. A random forest machine learning classifier was developed to distinguish between normal and malignant cells, and this classifier was subsequently used to predict the malignancy status of the remaining cells.

### Dimensional Reduction

PC analysis was conducted using the top 2000 highly variable genes. The number of PCs where the explained variance ratio exceeded 90% was determined, thus, the first 43 PCs were used for dimensional reduction. Uniform Manifold Approximation and Projection (UMAP) was employed to visually represent the distribution of individual cells while preserving the integrity of data structure and transcriptional similarity. In local approximations of the manifold structure, 30 neighboring points were utilized.

### Preprocessing and Annotation of Zebrafish scRNA‐seq Dataset

A zebrafish scRNA‐seq dataset with accession number GSE236393 was downloaded.^[^ ^25^ ^]^ To control the quality of the dataset, cells exhibiting fewer than 250 expressed genes and more than 6000 transcripts were excluded. Cells with mitochondrial RNA content exceeding 10% were also eliminated to mitigate the potential confounding effects of cellular stress or apoptosis. Additionally, features detected in fewer than three cells were removed from subsequent analyses. The filtered count data were then normalized and integrated using LogNormalize, FindIntegrationAnchros, and IntegrateData functions. To find out cell identities, PC analysis was implemented, and the first 50 PCs were applied to generate neighboring clusters using the FindClusters function. The cell compartments were identified using the canonical markers from the public database ZFIN (https://www.zfin.org/).^[^ ^55^ ^]^

### Drug Resistance Inference in scRNA‐seq Data

To characterize the drug responses according to the presence of *AP2M1* expression, a computational approach known as beyondcell was utilized.^[^ ^56^ ^]^ It identifies therapeutic differences between cell populations by calculating an enrichment score in a collection of drug signatures. Drug sensitivity signatures were collected, and the beyondcell score was computed using the GetCollection and bcScore functions. A lower score indicates a sensitivity to the drug, and a higher score indicates a resistance to the drug.

### Stemness Analysis in scRNA‐seq Data

The primitive potential was measured by leveraging the measure of transcriptional diversity using CytoTRACE2.^[^ ^57^, ^58^ ^]^ The implemented CytoTARCE2 v1.0.0 calculates the scores based on the biological principle that less differentiated cells tend to express a greater number of genes, making it an effective indicator that captures cellular multipotent potential. The score ranges from 0 to 1, with a higher score indicating a less differentiated status and higher stemness. In contrast, a lower score represents a more differentiated status, thus lower stemness.

### Scoring Enriched Signaling Pathway in scRNA‐seq Dataset

Enrichment score was computed using signaling pathways‐specific gene sets sourced from the KEGG pathway database.^[^ ^59^ ^]^ AUCell was employed to construct gene rankings based on the expression for each cell and calculate the area under the curve for each gene set.^[^ ^60^ ^]^ The enrichment scores were ranked based on the number of expressed genes and calculated within HSPC.

### Correlation Analysis

The correlation coefficient test using Spearman's method was applied to investigate the correlation between groups in the human blood samples and the scRNA‐seq data. In the analysis of scRNA‐seq data, the gene expression values obtained at the cellular level were aggregated and averaged based on cell type classification and donor identity.

### Statistical Analysis

The *t*‐test within the R environment was employed to analyze the statistical significance between groups. A significant level of *p*‐value was 0.05. The criteria for statistical significance were defined as follows: ^*^ *p* < 0.05, ^**^ *p* < 0.01, and ^***^ *p* < 0.001.

### Ethics Approval and Consent to Participate

This study received approval from the Institutional Review Board of Samsung Medical Center (approval No. 2023‐04‐094‐002). Informed consent was obtained from all subjects when the specimen was collected.

## Conflict of Interest

The authors declare no conflict of interest.

## Author Contributions

H.L., E.K.K., H.Y.J., and C.R.L. contributed equally to this work. K.S.J., K.H.Y., C.‐K.O., and Y.H.K. designed and supervised the study. H.L. performed the computational analyses. E.K.K. and C.R.L. performed the experiments. H.Y.J., K.H.Y., and K.S.J. obtained patient consent for the use of samples in the study. H.L., E.K.K., H.Y.J., C.R.L., K.S.J., K.H.Y., C.‐K.O., and Y.H.K. wrote the manuscript, prepared figures, and discussed the results. S.A., E.S.K., K.M., W.K.K., S.‐Y.C., Y.K., D.K., E.J.K., Y.Y, H.K., S.I.M., D.M.L., K.K., H.C., E.Y.C., T.S.G., N.B., and D.L. discussed the results. All authors have read and approved the final version of the manuscript.

## Supporting information



Supporting Information

## Data Availability

The data that support the findings of this study are openly available in Gene Expression Omnibus at 10.1093/nar/gki022 [61]. These data were derived from the following resources available in the public domain, GSE236393, https://www.ncbi.nlm.nih.gov/geo/query/acc.cgi?acc = GSE236393, GSE116256, https://www.ncbi.nlm.nih.gov/geo/query/acc.cgi?acc = GSE116256.
