# Supplementary material for: AP2M1 Amplification Orchestrates Notch‐Mediated Chemoresistance in Hematopoietic Stem Cells of Acute Myeloid Leukemia Patients
Source: Adv Sci (Weinh). 2025 Oct 13;12(48):e14566. doi: 10.1002/advs.202514566 (PMC12752594; doi:10.1002/advs.202514566)
Supplement: Supplementary file 1 — Supporting Information [file ADVS-12-e14566-s001.docx]

**Supplementary information**

AP2M1 Amplification Orchestrates Notch-Mediated Chemoresistance in

Hematopoietic Stem Cells of Acute Myeloid Leukemia patients

*Corresponding author:

Ki Sun Jung, M.D, Ph.D.,

Department of Internal Medicine, Pusan National University Yangsan Hospital, Pusan National University School of Medicine, Yangsan, 50612, Republic of Korea, Tel: 82-51-1577-7512, Email: ks114-jung@naver.com

Keon Hee Yoo, M.D, Ph.D.,

Department of Pediatrics, Samsung Medical Center, Sungkyunkwan University School of Medicine, Seoul, 06351, Republic of Korea, Tel: 82-1599-3114, Email: hema2170@skku.edu

Chang-Kyu Oh, M.D, Ph.D.,

Department of Biochemistry, School of Medicine, Pusan National University, 49 Busandaehak-ro, Yangsan, **50612**, Republic of Korea, Tel: +82-51-510-8083, Email: [ck1988@pusan.ac.kr](mailto:ck1988@pusan.ac.kr), and

Yun Hak Kim, MD, PhD.

Department of Anatomy and Department of Biomedical Informatics, School of Medicine, Pusan National University, 49 Busandaehak-ro, Yangsan, 50612, Republic of Korea, Tel: +82-51-510-8091, Email: [yunhak10510@pusan.ac.kr](mailto:yunhak10510@pusan.ac.kr)

**Supplementary figures**

**Supplementary Figure 1**


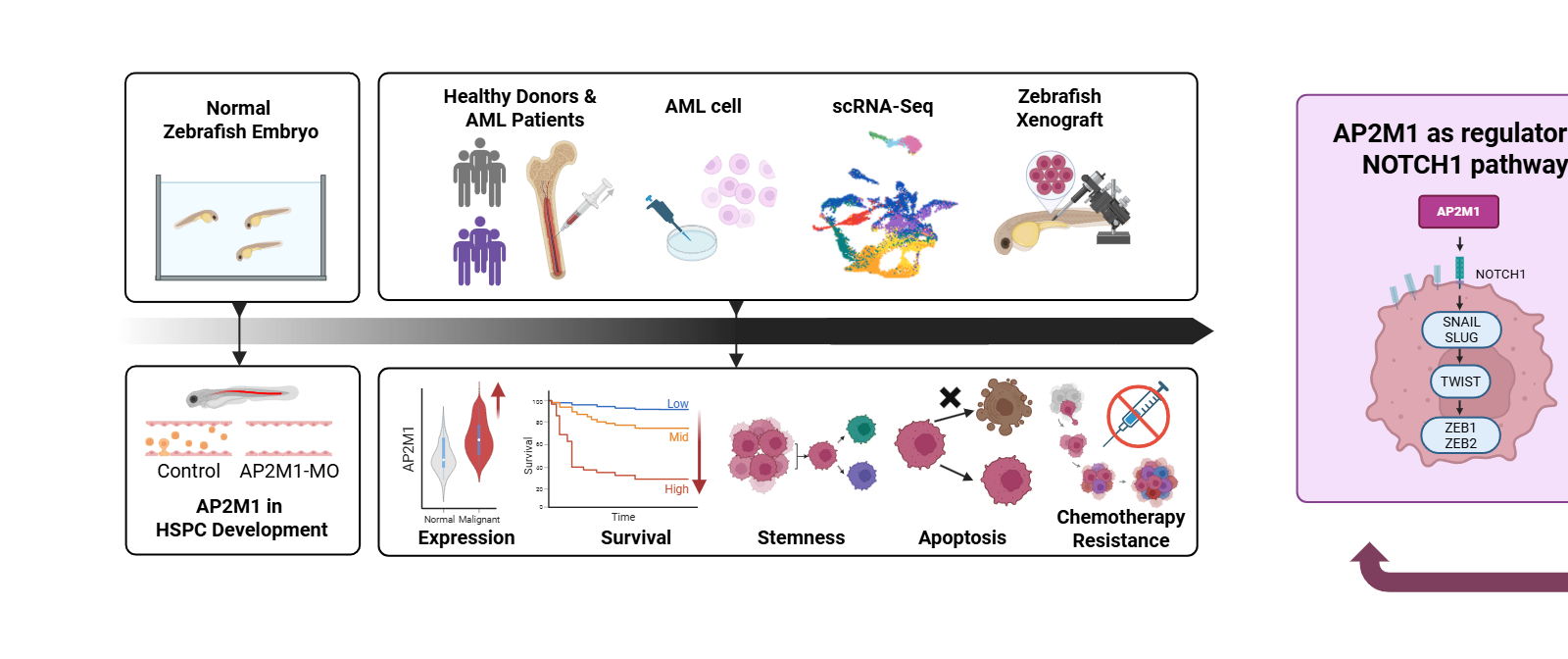


Schematic workflow of this study. The role of AP2M1 in normal hematopoiesis was initially investigated using zebrafish embryo models. Subsequently, AP2M1 expression levels and their association with clinical outcomes were evaluated by analyzing bone marrow samples with single-cell RNA sequencing (scRNA-seq) datasets from both healthy donors and patients with acute myeloid leukemia (AML). The influence of AP2M1 on stemness, apoptosis, and chemotherapy resistance in AML cells was assessed through a comprehensive approach incorporating multiple data sources, including bone marrow specimens, AML cell lines, scRNA-seq datasets, and zebrafish xenograft models.

**Supplementary Figure 2**
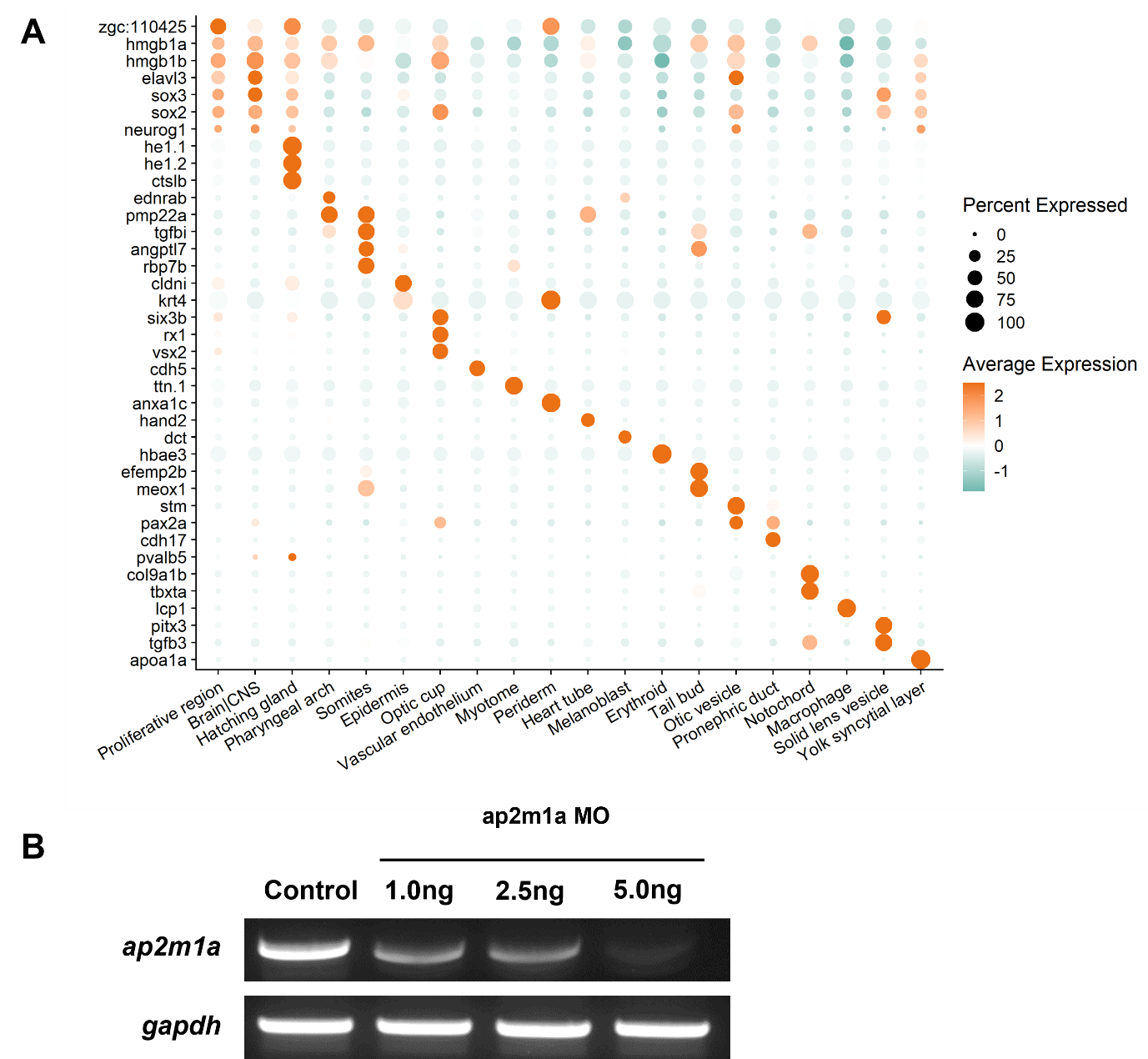


1. Canonical markers for cell annotation in scRNA-seq data of zebrafish. The color of the dots represents the gene expression level, while the size of the dots illustrates the percentage of cells expressing the gene.
2. RT-PCR data of *ap2m1a* expression in zebrafish embryos injected with *ap2m1a* morpholino (MO) at concentrations of 1.0ng, 2.5 ng and 5.0 ng.

**Supplementary Figure 3
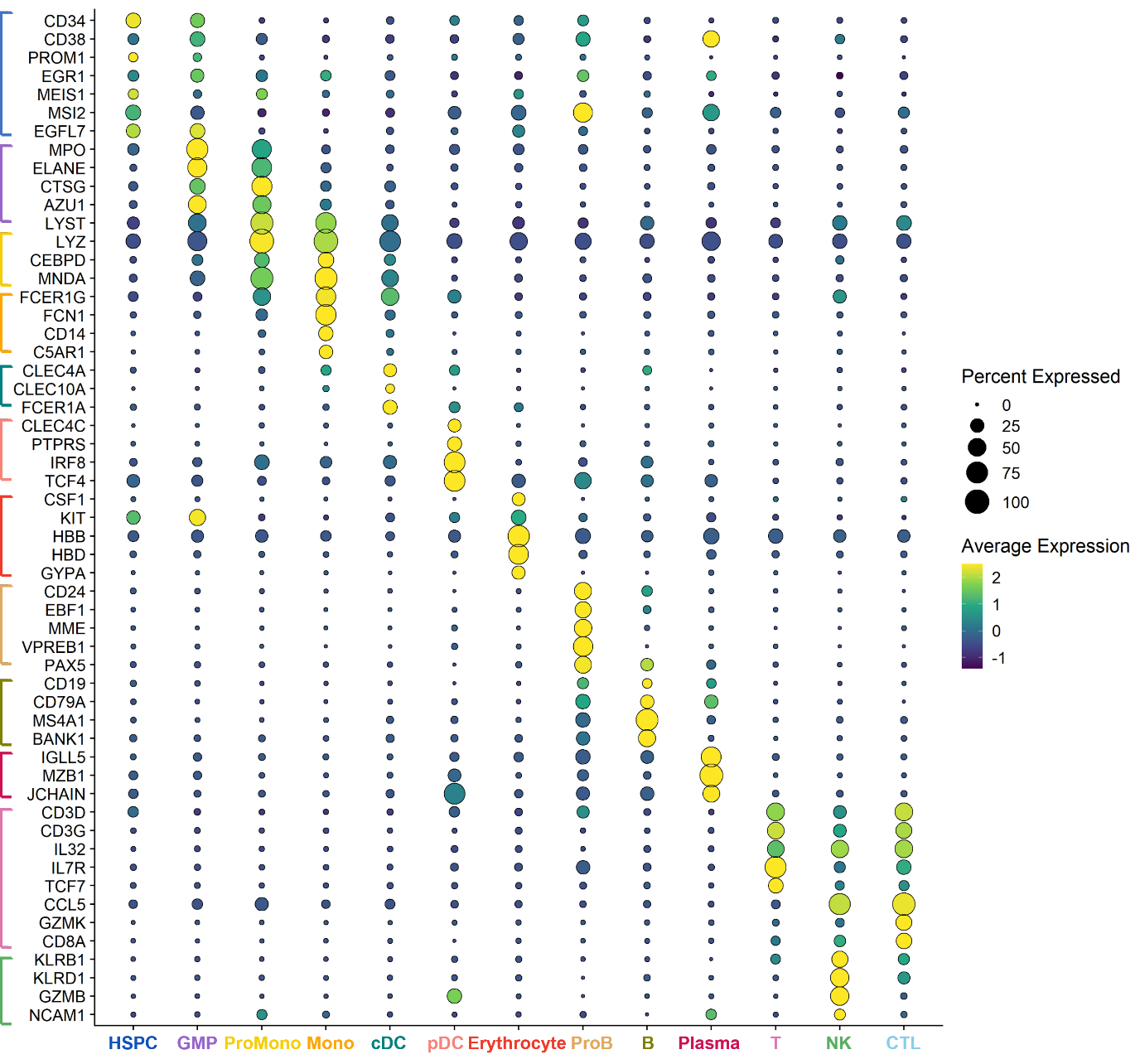
**

Canonical markers for cell annotation in scRNA-seq data of AML dataset. The color of each cell type on the x-axis matches the color of the angle brackets on the y-axis that group the corresponding markers.

**Supplementary Figure 4**

**
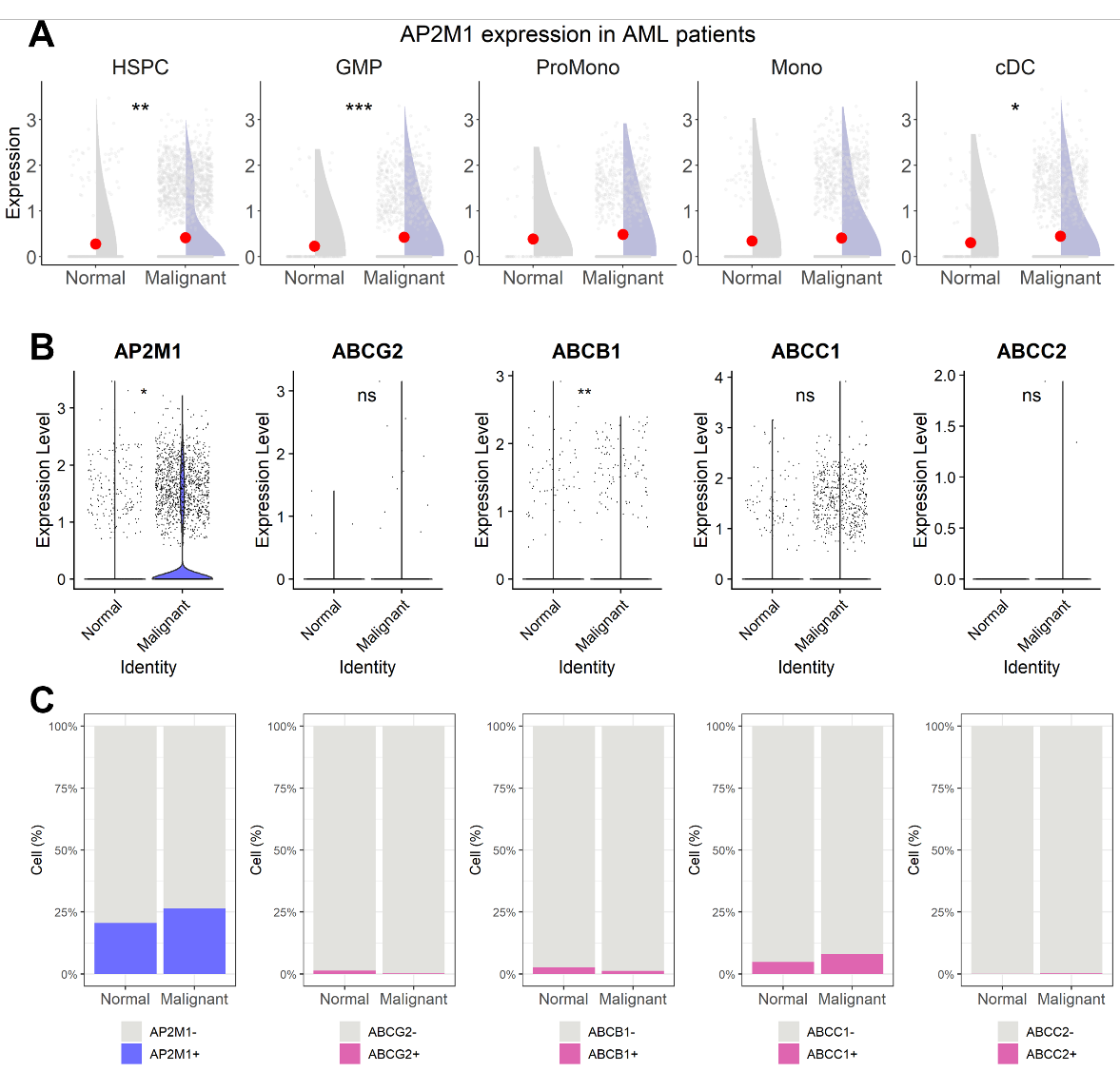
**

**A.** Expression level of *AP2M1* for each cell type within AML patients. *p < .05, **p < 0.01, ***p < .001.

**B.** Expression level of *AP2M1* and ABC transporter genes in HSPCs.

**C.** Gene-expressing cell percentage depending on the malignancy in HSPCs.

**Supplementary Figure 5**

**
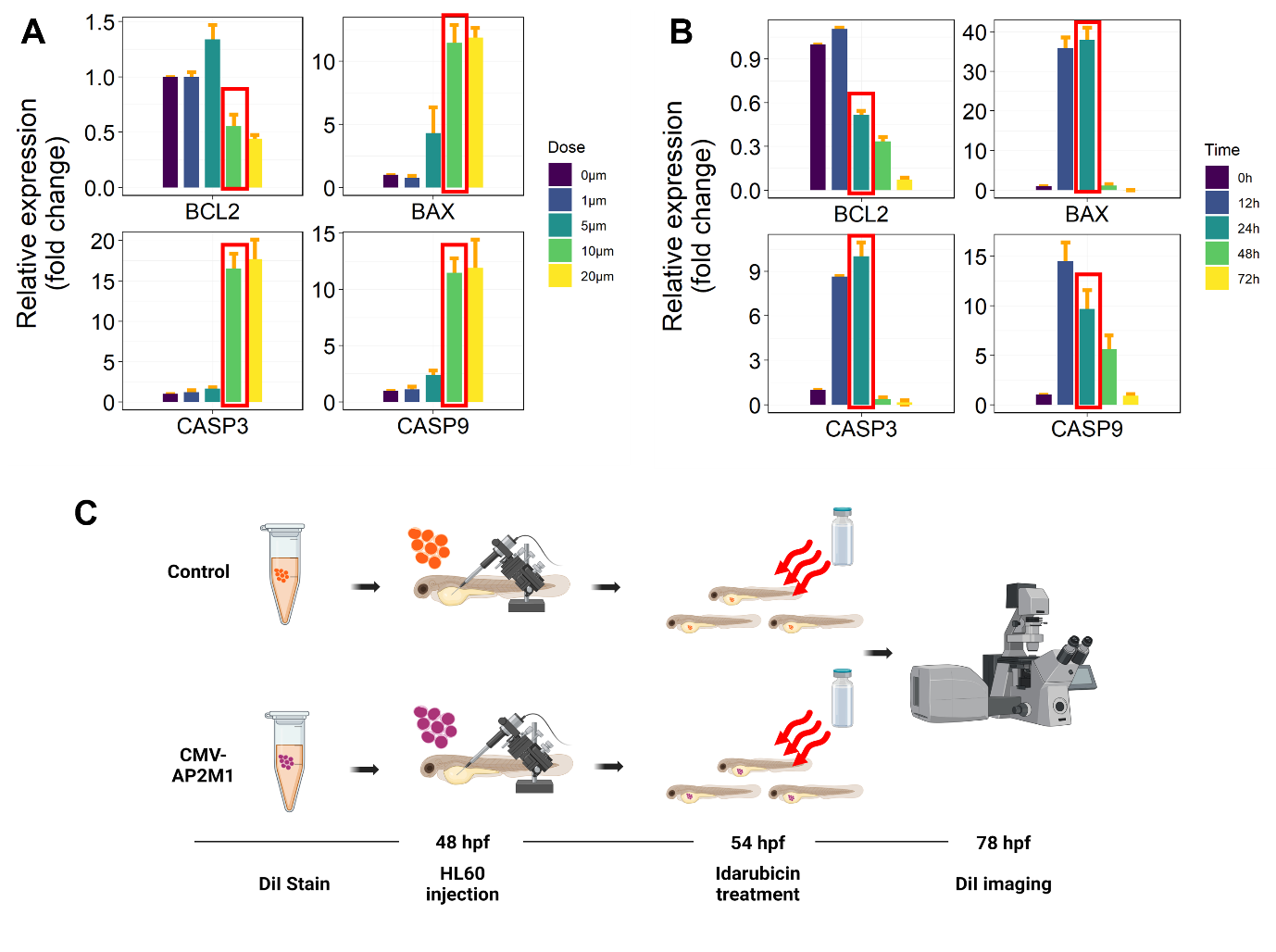
**

1. The mRNA level change of apoptosis-associated genes. The levels were measured based on varying doses of Idarubicin (A) and over time with a 10 µM concentration of the drug (B). Diagram for the process of DiI imaging. The *ap2m1a* cells and CMV- *ap2m1a* cells were stained with DiI and injected into 48 hpf zebrafish embryos, followed by idarubicin treatment at 54 hpf. The cells were observed via fluorescence imaging (C).

**Supplementary Figure 6**

**
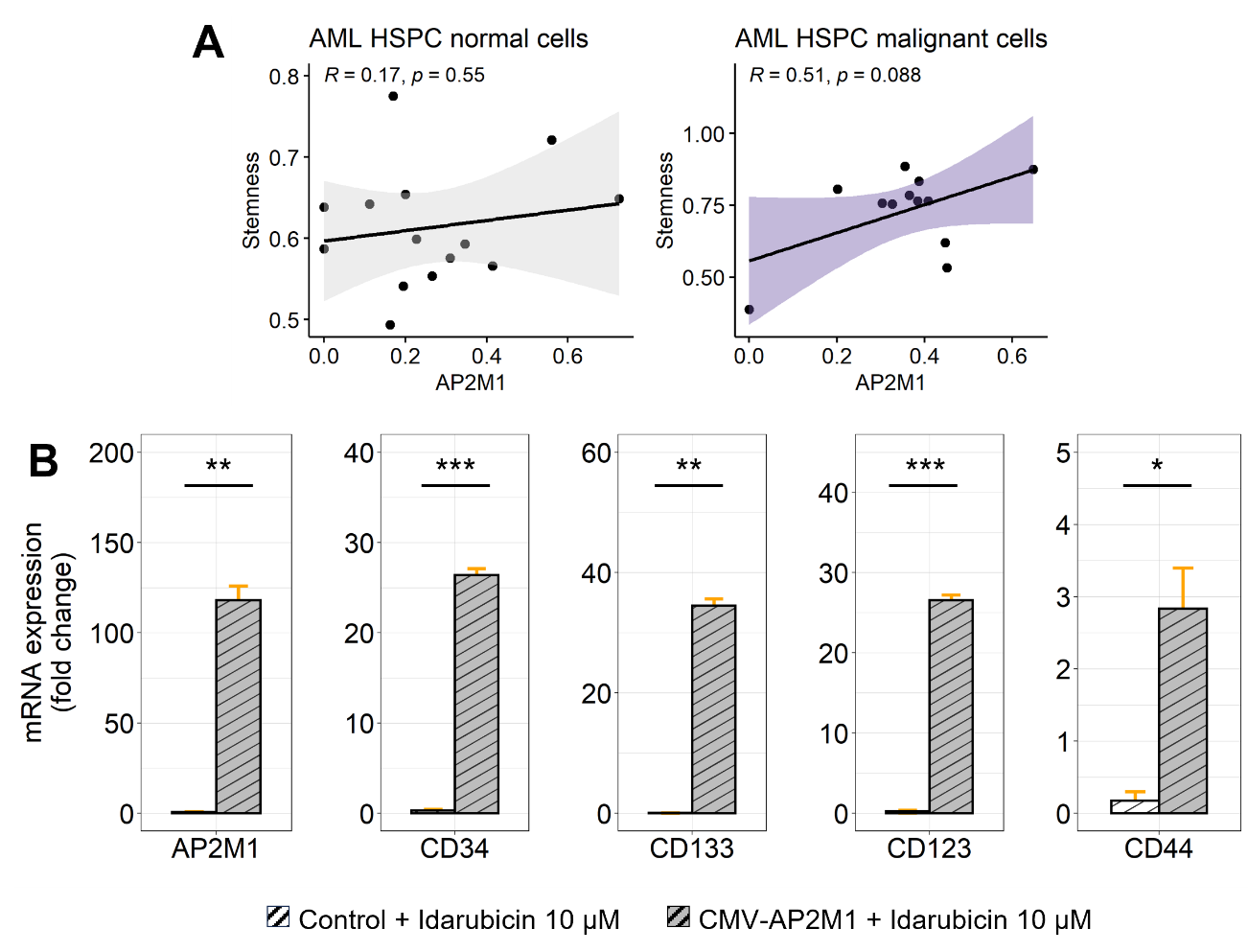
**

**A.** Association between *AP2M1* and stemness score in HSPC cells from AML patients. The relationship was explored according to the malignancy of HSPC cells, and each dot represents an individual AML patient.

**B.** The mRNA expression level of stemness-related genes treated by Idarubicin. The levels are measured in control and AP2M1 overexpressed cell lines. *p < .05, **p < 0.01, ***p < .001.

**Supplementary Figure 7**

**
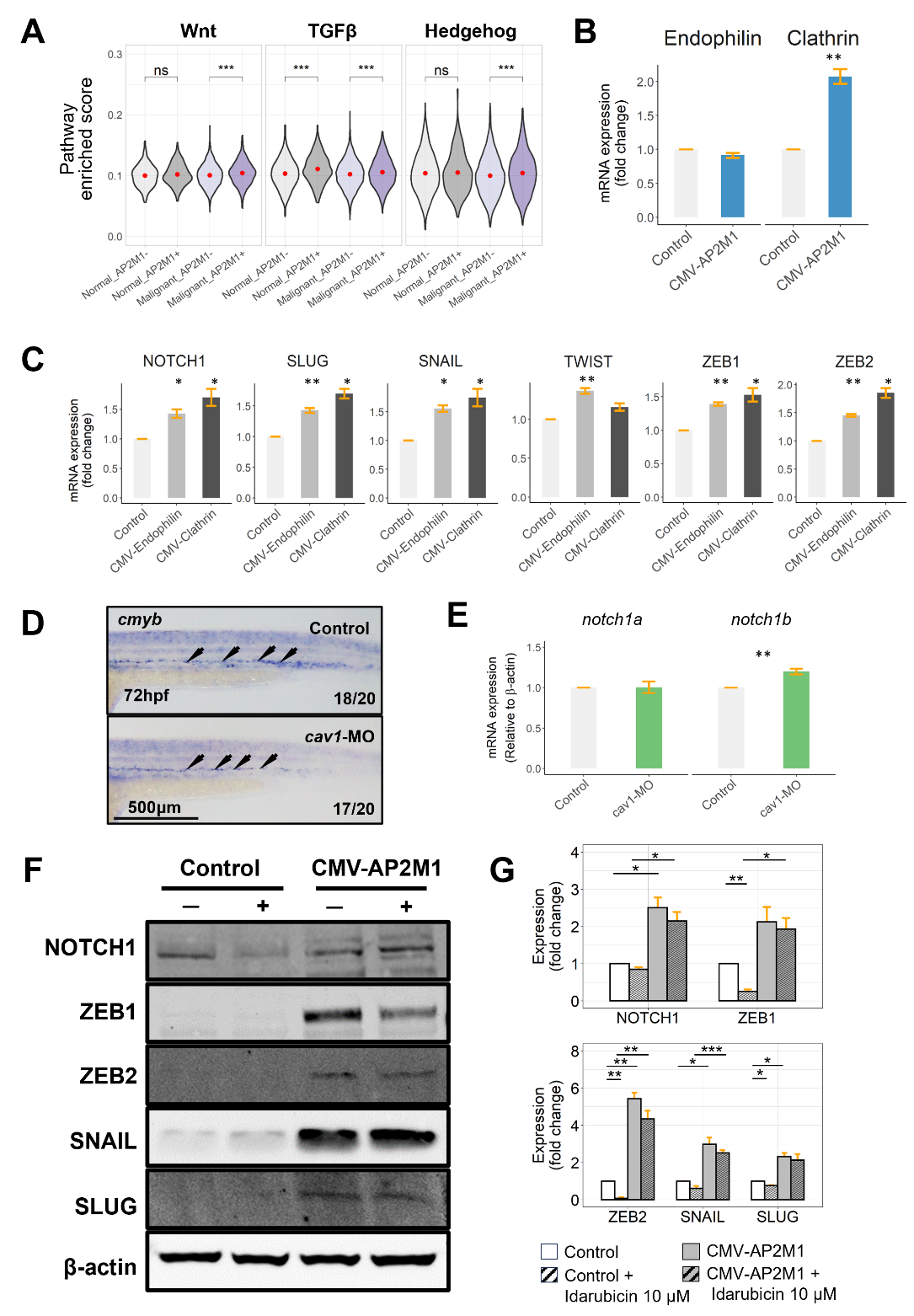
**

A. The enriched score of Wnt, TGFβ, and Hedgehog signaling pathway according to cell malignancy and *AP2M1* expression in HSPCs. *p < .05, **p < 0.01, ***p < .001

B. Analysis of endophilin and clathrin mRNA levels in AP2M1-overexpressing cells. mRNA expression of endophilin and clathrin was measured by qRT-PCR in control and AP2M1-overexpressing cells.

C. Relative mRNA expression levels of Notch signaling target genes comparing control and cells overexpressing endophilin and clathrin, respectively.

D. WISH analysis and lateral view imaging of *cmyb* expression in the CHT of 72 hpf zebrafish embryos injected with 2.5 ng/nL of WT and caveolin1-MO zebrafish embryos.

E. qPCR analysis of *notch1a* and *notch1b* mRNA expression level in *caveolin1* knockdown zebrafish embryos.

F-G. Assays evaluating changes in the downstream genes of the Notch signaling. These alterations were assessed in both control and AP2M1-overexpressing cells, as well as following drug treatment, at protein (F) and mRNA levels (G).
